# Acid Conditions of the Fluids of the Mouth

**Published:** 1877-03

**Authors:** 


*Acid Condition of the Fluids of the Mouth.*—Dr. Hodson in the *Medical Record*, notices the fact that in any illness causing a feverish condition of the mouth, the oral fluids are intensely acid, and affect the teeth to the same degree as does any acid medicine administered in practice; and also, that from the high temperature of the buccal cavity at such times the power of these acids is greatly increased.

A direct consequence of these conditions is the very rapid fermentation of food lodged abont the teeth, as well as its decomposition, and the consequent elimination of other deleterious acids. Lime water, *liquor calcis*, diluted according to the sensitiveness of the mucous membrane of the mouth, and flavored with a few drops of wintergreen or peppermint to make it agreeable, is recommended to correct this acid condition of the oral fluids by its anti-acid properties.

